# A Mechanism-Based Classification and Review of Endoscopic and Ultrasound-Guided Carpal Tunnel Release

**DOI:** 10.3390/jcm15166130

**Published:** 2026-08-07

**Authors:** Malka Korenblit, Evan J. Haas, Elena M. Esch, Lynn Orfahli, Olivier Mares, Mark Greyson

**Affiliations:** 1 Division of Plastic and Reconstructive Surgery, Department of Surgery, University of Colorado Anschutz, Medical Campus, 12631 E 17th Ave, Aurora, CO 80045, USA; malka.korenblit@cuanschutz.edu (M.K.);; 2 Service de Chirurgie Orthopédique et Traumatologique, Centre Hospitalo-Universitaire de Nîmes, Avenue du Pr. Debré, 30000 Nîmes, France

**Keywords:** carpal tunnel release, carpal tunnel syndrome, ultrasound, endoscopic, hand surgery, peripheral nerve, minimally invasive carpal tunnel release, WALANT

## Abstract

**Background**: Minimally invasive carpal tunnel release (CTR) approaches, including endoscopic and ultrasound-guided, encompass a range of distinct techniques. These approaches have been increasingly adopted across both surgical and non-surgical specialties. However, the literature frequently groups the techniques into broad categories, obscuring clinically meaningful differences in transection mechanism, safety profile, and learning curve. **Methods**: This narrative review organizes minimally invasive CTR into eight distinct techniques, two endoscopic and six ultrasound-guided, classified by their primary transection mechanism. **Results**: Single-portal endoscopic CTR has the largest evidence base, with network meta-analysis data demonstrating significant early functional advantages over open CTR. Among ultrasound-guided techniques, the retractable retrograde blade is the most widely adopted, with randomized trial data supporting comparable outcomes to mini-open CTR. Hook-knife retrograde and thread-loop techniques have demonstrated feasibility and durable outcomes, though experience is concentrated among a few high-volume operators. The antegrade cutting, pinhole scalpel, and needle-knife have more limited evidence. Earlier return to work is the most reproducible benefit across all minimally invasive approaches. Endoscopic CTR carries a modestly higher revision rate, while long-term data for ultrasound-guided techniques remain lacking. **Conclusions**: This mechanism-based classification highlights important differences between techniques in safety features, evidence maturity, and learning curves. Head-to-head trials comparing endoscopic and ultrasound-guided techniques and device-specific registries with standardized outcome reporting are needed to inform evidence-based adoption.

## 1. Introduction

Carpal tunnel syndrome is the most common compressive peripheral neuropathy of the upper extremity, with a prevalence of approximately 1–5% in the general population [1,2]. While conservative management, including splinting and corticosteroid injection, offers temporary symptom relief, surgical release remains the definitive treatment for moderate-to-severe or refractory carpal tunnel syndrome [2,3]. Carpal tunnel release (CTR) is among the most frequently performed hand surgeries worldwide, with approximately 350,000 to 500,000 procedures performed per year in the United States alone [4].

Open and mini-open CTR have been the standard surgical approaches, providing direct visualization of the transverse carpal ligament (TCL) and surrounding structures. Two minimally invasive approaches have since emerged. Endoscopic CTR (ECTR), introduced in the late 1980s, achieves ligament transection through one or two small incisions under endoscopic visualization [5,6,7]. Ultrasound-guided CTR (UGCTR), first described in 1997, uses real-time sonographic visualization to transect the ligament through a small wrist or palmar incision [8]. Both ECTR and UGCTR have been performed safely in office-based settings, and the growing number of commercially available devices and procedural modifications reflects increasing clinical interest [9].

Despite this expanding landscape, the literature frequently groups ECTR and UGCTR into broad categories, obscuring clinically meaningful differences between individual techniques. These approaches employ distinct transection mechanisms with differing safety profiles, learning curves, and practice-setting requirements. As minimally invasive CTR is increasingly adopted across both surgical and non-surgical specialties, classification is needed to guide technique selection and future research. This review organizes minimally invasive CTR into eight distinct techniques, two endoscopic and six ultrasound-guided, classified by their transection mechanism. For each, we describe the technique, summarize available evidence, and discuss practical considerations relevant to clinical adoption.

We chose the transection mechanism as the primary organizing principle because it determines the biomechanical interaction between the cutting instrument and the TCL, which in turn governs the safety features available to the operator, the direction of force relative to at-risk structures, and the learning curve. Classifying by surgical approach (endoscopic vs. ultrasound-guided) obscures the fact that techniques within each category differ fundamentally in how the ligament is divided. Classifying by commercial device risks obsolescence as new products enter the market and conflates engineering differences with mechanistic ones. A mechanism-based framework is therefore intended to be durable across device iterations and to facilitate head-to-head comparisons between techniques that share a common transection principle.

## 2. Methods

This narrative review was conducted by searching PubMed/MEDLINE, Embase, and the Cochrane Library from inception through June 2026 using the terms ‘carpal tunnel release’ combined with ‘endoscopic,’ ‘ultrasound-guided,’ ‘percutaneous,’ and individual device or technique names. Reference lists of included studies, recent meta-analyses, and clinical practice guidelines were hand-searched for additional relevant publications. Studies were selected based on relevance to the classification framework, prioritizing randomized controlled trials, meta-analyses, and multicenter cohorts where available. As a narrative review, no formal risk-of-bias assessment or systematic quality grading was performed.

## 3. Anatomy and Safe-Zone

The TCL forms the roof of the carpal tunnel, attaching to the scaphoid tubercle and trapezium radially and the pisiform and hook of the hamate ulnarly. The structures at risk are the median nerve and its recurrent motor branch, the palmar cutaneous branch of the median nerve, the ulnar neurovascular bundle in Guyon canal, the superficial palmar arch, and the Berrettini communicating branch [10].

Minimally invasive approaches rely on identifying two orthogonal safe zones through which the blade can be advanced beneath the TCL without violating critical structures. The transverse safe zone is the interval between the ulnar artery (or hook of the hamate) and the median nerve, approximately 6 mm in neutral wrist position and widened by passive wrist deviation [11,12]. The longitudinal safe zone is the interval between the distal edge of the TCL and the superficial palmar arch, which lies approximately 12–19 mm distal to the distal TCL edge [13]. For single-portal endoscopic techniques, the operative safe zone is the triangle bounded by the ulnar half of the distal TCL edge, the ulnar border of the median nerve, and the proximal superficial palmar arch margin [5,14]. The apex of the hook of the hamate serves as a sonographic landmark for the distal TCL, lying approximately 11–12 mm proximal to it across the transverse safe zone [15].

Clinically relevant median nerve variants include a transligamentous recurrent motor branch (~11% prevalence), bifid median nerves (~3%; often co-occurs with persistent median artery), and distal or proximal accessory branches (~2–5%) [16,17]. The Berrettini communicating branch is present in approximately 61% of hands and may lie within the distal release zone [18,19]. Anomalous lumbrical muscles or an accessory abductor digiti minimi within the tunnel can reduce the intratunnel space available for instrument passage and impair sonographic visualization [20].

The transligamentous branch pierces the TCL and lies directly within the plane of ligament division, making it the variant most exposed during minimally invasive CTR. Its pooled prevalence is approximately 11% but is strongly linked to the presence of a hypertrophic muscle overlying the distal TCL: a transligamentous course is found in 23.4% of hands with such a muscle versus 1.7% without [16]. In a prospective series (*n* = 100), every transligamentous and preligamentous branch was associated with this anomalous overlying musculature [21]. This hypertrophic muscle and the motor branch course are reliably identified on high-frequency ultrasound, with excellent agreement to cadaveric dissection [22].

## 4. Endoscopic CTR

### 4.1. Single-Portal

Single-portal ECTR, introduced by Agee in 1992, uses an integrated blade and endoscope, allowing the operator to simultaneously visualize and cut the ligament through a single portal. The device, commonly the SmartRelease (MicroAire Surgical Instruments, Charlottesville, VA, USA), is introduced through a transverse wrist-crease incision (typically 1–2 cm) and advanced distally into the carpal canal [5]. The trigger-activated blade is elevated against the deep surface of the ligament, and the device is withdrawn proximally, transecting the TCL in a retrograde (distal-to-proximal) fashion [5]. Single-portal systems using an antegrade transection mechanism have also been described (SEGway System, SEGway Orthopaedics, Carlsbad, CA, USA) [23].

Single-portal ECTR has the largest evidence base of any minimally invasive technique, with multiple RCTs and meta-analyses. Early multicenter randomized controlled trials (RCTs) demonstrated early recovery of grip strength and shorter return to work (18 days versus 38 days for open), with equivalent one-year outcomes [24]. In a 2025 network meta-analysis comparing seven CTR techniques, single-portal ECTR showed significant reductions in symptom severity at one, three, and six months, and the largest functional improvement at all timepoints compared to open CTR [25]. Pooled intraoperative conversion to open CTR across 78 series (40,351 cases) was 0.62%, most commonly for hypertrophic tenosynovium or aberrant median nerve anatomy; rates of conversion to open fall sharply with experience [26,27].

### 4.2. Dual-Portal

Dual-portal ECTR, introduced by Chow in 1989, is performed through two incisions, allowing the endoscope and cutting instrument to be introduced from opposite ends. A proximal transverse incision (~1 cm) just radial to the pisiform and a distal mid-palm incision are made; a trocar and endoscopic cannula are advanced from proximal to distal beneath the TCL with the wrist and fingers hyperextended. The endoscope is inserted through the distal portal to visualize the deep surface of the TCL, while a hook knife or probe-knife is introduced through the proximal wrist portal and transects the TCL in a retrograde (distal-to-proximal) fashion [6].

Randomized comparison with open CTR shows less scar tenderness and faster return to work, with equivalent longer-term outcomes; early safety concerns prompted refinement of intraoperative technique before widespread adoption [28]. The 2025 network meta-analysis demonstrated superior symptom relief compared with open CTR at three months and reduced pain at one and three months with dual-portal ECTR compared with open. Despite the theoretical advantage of bidirectional visualization, indirect comparisons show no significant safety or efficacy differences between dual- and single-portal ECTR [29].

## 5. Ultrasound-Guided CTR

**Figure 1:**
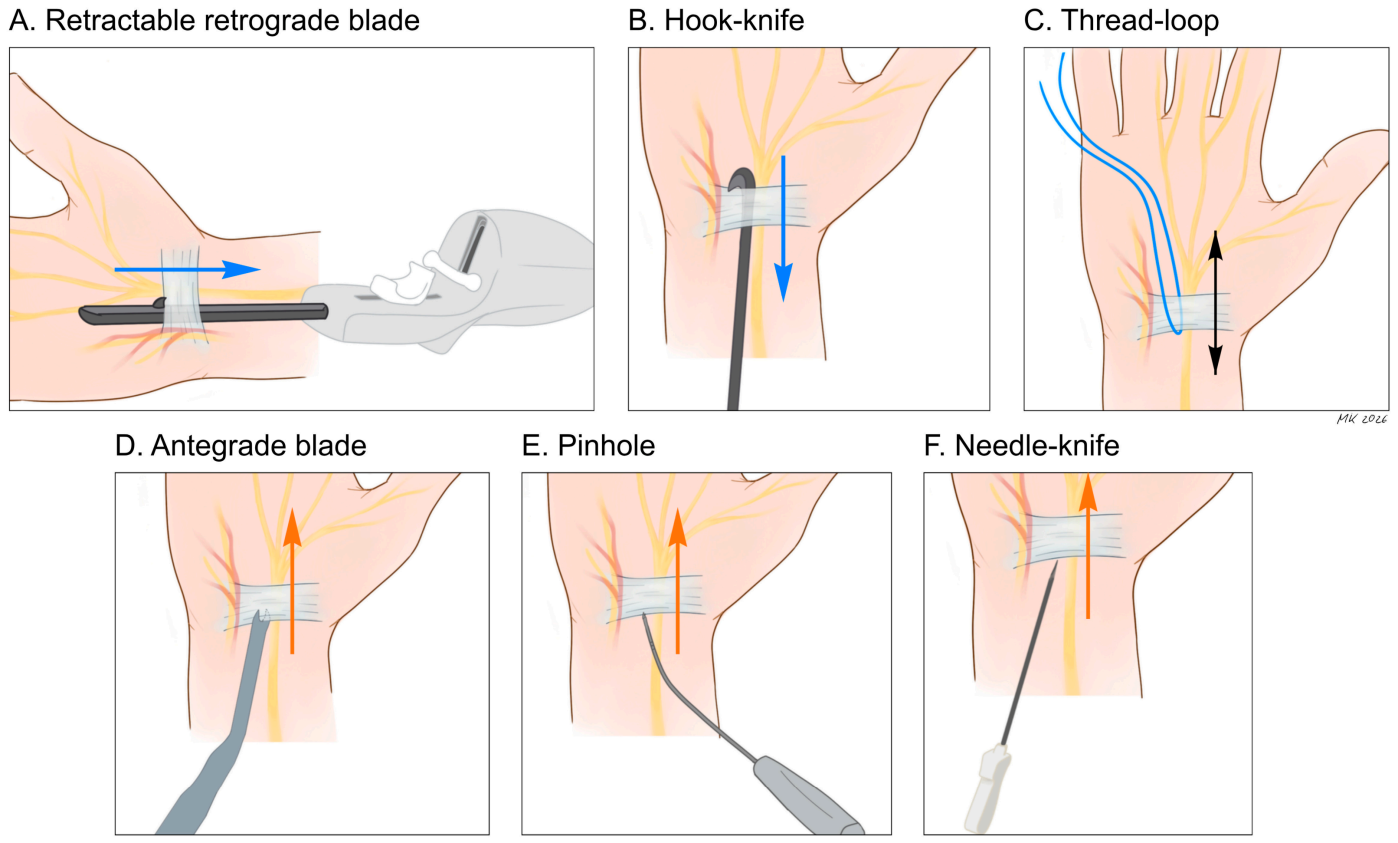
Schematic illustrations of ultrasound-guided carpal tunnel release mechanisms, demonstrating device positioning relative to the TCL and median nerve. Yellow structures represent the median nerve and its branches, red structures represent the ulnar artery. The gray band represents the transverse carpal ligament. Arrows indicate the cutting direction (blue = retrograde; orange = antegrade; black = bidirectional).

Figure 1 illustrates the transection mechanisms of four ultrasound-guided techniques.

### 5.1. Retractable Retrograde Blade

The retractable retrograde blade is introduced through a sub-centimeter distal forearm incision at the level of the lunate and advanced through the carpal canal after initial hydrodissection [30]. A saline-inflated balloon within the device is deployed to displace the median nerve and flexor tendons volarly and radially while elevating the TCL into a defined cutting plane, creating a protected safe zone. The TCL is then transected in a single retrograde pass using a retractable blade. In cases of very thick ligaments, more than one pass may be utilized, either to complete the transection or to create initial space allowing for greater displacement of the nerve away from the cutting area.

This is the most widely adopted UGCTR mechanism in the United States. Evidence within this class is derived primarily from studies of the proprietary single-use SX-One MicroKnife (Sonex Health, Eagan, MN, USA). The industry-funded multicenter TUTOR RCT (SX-One MicroKnife vs. mini-open; *n* = 149) showed comparable patient-reported outcomes and functional improvement with smaller incisions and substantially less scar-related morbidity [30,31]. A single-center retrospective series reported sustained satisfaction (93%) at a median of 1.7 years [32]. Multicenter manufacturer-sponsored registry data have demonstrated feasibility with a median return to work of 3–4 days and a 0.4% complication rate, though registry designs lack control groups and independent adjudication [33].

Related devices in this class have the same primary transection mechanism but lack a discrete safety mechanism. The published clinical experience for the MICROi-Blade (Summit Medical Products, Inc, Sandy, UT, USA), a needle-mounted retractable blade, consists of a 166-case series by the device’s inventor with no reported complications at 3–6 months [34]. The MANOS CTR™ (Thayer Intellectual Property, Inc., San Francisco, CA, USA), originally described as a nerve-stimulator-guided device, has been adapted for ultrasound guidance [35,36].

### 5.2. Hook-Knife

Hook-knife retrograde CTR transects the TCL using a small curved-hook blade under continuous ultrasound guidance [37]. The device is introduced through a sub-5 mm distal antebrachial incision and advanced beneath the ligament to the distal TCL edge. This technique relies on hydrodissection as its primary safety mechanism; local anesthetic injected into the canal separates the median nerve volarly from the deep TCL surface, creating a longitudinal safe zone for blade passage. The hook is then rotated to engage the ligament and pulled retrograde to transect it [38,39]. Some operators add a blunt button-tip guiding cannula for atraumatic dilation and repeat hydrodissection [37].

Evidence within this class is derived primarily from studies on the Acufex^®^ 3.0 mm hook-knife (Smith & Nephew PLC, London, UK). A single-center RCT (hook-knife vs. mini-open; *n* = 92) demonstrated substantially faster functional recovery and equivalent neurologic improvement [38]. A prospective single-operator series (*n* = 129) demonstrated complete TCL transection on magnetic resonance imaging at one month, including in patients with bifid median nerves, with no reported complications [40]. Published experience is concentrated in a small number of high-volume operators with no multicenter cohorts.

### 5.3. Thread-Loop

Thread-loop CTR transects the TCL by reciprocating a percutaneously looped cutting thread under continuous ultrasound, requiring two needle puncture points and no skin incision. An epidural needle (typically a pre-bent 18G Tuohy, Perifix (B. Braun, Bethlehem, PA, USA), BD (Becton, Dickinson and Company, Skokie, IL, USA), and (Myco Medical, Apex, NC, USA)) is routed from a palmar entry through the carpal canal to a wrist exit, first deep to the TCL and then superficial to it, with hydrodissection at each pass; a fine cutting thread (Loop&Shear, Monterey Park, CA, USA or Smartwire, Ilsan, South Korea) is then looped around the ligament and drawn back and forth until the TCL is divided [41,42].

Cadaveric validation confirmed complete TCL transection without neurovascular damage [43]. A subsequent 159-wrist clinical series demonstrated significant symptom improvement with most office workers returning to work on postoperative day 1 [41]. These findings were supported by a small RCT (*n* = 20) demonstrating significant improvements in symptom and functional scores, distal motor latency, and transcarpal conduction velocities at 6 months with no complications and 85–90% patient satisfaction [44]. A prospective intrasubject-randomized trial (*n* = 11) showed no difference in functional outcomes between sequential thread-loop and mini-open CTR at 12 months; 10 of 11 patients preferred thread-loop CTR, citing faster recovery and less pain [45]. A retrospective cohort (82 wrists) reported durable symptom relief at two years and no recurrence, though long-term data from larger, multicenter studies are not yet available [46].

### 5.4. Antegrade Blade

Antegrade cutting techniques transect the TCL by advancing a curved blade in a proximal-to-distal direction under continuous ultrasound guidance, with an integrated or separate guiding element to constrain the cutting path. The blade is introduced through a small wrist-crease incision (typically 7–15 mm) and applied to the proximal edge of the TCL; continuous forward pressure is applied to divide the full length of the ligament. Hydrodissection separates the median nerve from the deep surface of the TCL before blade insertion.

Evidence within this class is derived from two proprietary devices. The KeriKnife (KeriMedical, Plan-les-Ouates, Switzerland) has an integrated cutting guide positioned deep to the TCL to isolate the median nerve from the cutting path, a distal head element halts the knife to prevent over-advancement and protect the superficial palmar arch [47]. However, the protective guide has undergone redesign due to volumetric constraints within the carpal canal, and the current iteration relies primarily on hydrodissection and ultrasound visualization for nerve protection. The Kemis H3 (Newclip Technics, Haute-Goulaine, France) does not incorporate a separate guiding cannula, and safety relies on a protective end tip [48]. A cadaveric study demonstrated 98% complete TCL transection using the KeriKnife [47]. A prospective observational cohort study (*n* = 50; 25 percutaneous, 25 open) using the Kemis H3 demonstrated no statistically significant differences in functional scores or complication rates compared to open CTR. Patients in the Kemis H3 group recovered grip strength faster at 6 weeks, though this difference equalized by 3 months [48]. Neither device has been evaluated in an RCT, and no multicenter or long-term data exist. Published evidence for both includes inventor or manufacturer involvement.

Although the KeriKnife and Kemis H3 share an antegrade transection mechanism and are therefore grouped within the same class, they differ in blade geometry, guiding-element design, and safety features, which may influence handling characteristics, procedural workflow, and the learning curve. These engineering distinctions merit independent evaluation as clinical data mature.

### 5.5. Pinhole Scalpel

The pinhole scalpel (Sono-Instruments; Spirecut AG, Muttenz, Switzerland) divides the TCL under mechanical tension, theoretically reducing the risk of injury to adjacent non-tensed soft tissues [49]. A 1.5 mm cutter, designed for enhanced visibility under ultrasound, is introduced through a single 14-gauge catheter puncture and advanced antegrade on the ulnar side of the canal, within the safe zone between the hook of the hamate and the median nerve, and divides the TCL through progressive cuts [49,50].

A single-surgeon pre-CE-marking prospective series (*n* = 30) demonstrated successful TCL transection in all cases, no adverse events, and significant QuickDASH improvement at two months; however, 53% of patients reported pillar pain at two months [50]. The evidence base is limited to the developer-surgeon’s practice, with disclosed financial interests, and the tension-selective mechanism has not been independently validated.

### 5.6. Needle-Knife (Acupotomy/Miniscalpel-Needle)

Needle-knife UGCTR uses a flat-bladed instrument shaped like an acupuncture needle (miniscalpel-needle or acupotomy, 小针刀) with a small, sharp cutting edge at the tip. The TCL is divided by direct incision, with safety dependent on the operator’s ultrasound visualization. The needle is inserted under continuous ultrasound at a shallow angle from the ulnar side of the median nerve and advanced through the TCL; the ligament is transected by forward-and-backward passes [51,52].

Meta-analyses of predominantly small, single-center RCTs suggest improved functional outcomes and clinical cure rate compared to conservative treatment, non-ultrasound-guided needle-knife, and mini-open CTR [53,54]. Significant heterogeneity limits the strength of these conclusions. The evidence base is concentrated in East Asian practice settings, and multicenter validation in other populations is lacking.

## 6. Synthesis of Comparative Evidence

Table 1 summarizes the key features, safety mechanisms, and evidence maturity for each of the eight minimally invasive CTR techniques described above.

### 6.1. ECTR vs. UGCTR

A prospective multicenter propensity-matched cohort (*n* = 372; ECTR vs. UltraGuideCTR retractable blade) found both approaches produced large, comparable improvements in symptoms, function, and quality of life at three months, with between-group differences below clinically meaningful thresholds [55]. The principal distinctions were that periprocedural UGCTR was more often performed under WALANT, with lower postoperative opioid use (10.3% vs. 39.7%) and higher overall satisfaction (92.1% vs. 83.6%) and wound satisfaction, whereas ECTR had a shorter procedure time (8 vs. 17 min). Nonserious adverse events were less frequent after UGCTR (0% vs. 5.9%), with no serious events in either arm. This cohort was industry-funded and observational; endoscopic surgeons were more experienced, and follow-up is limited to three months [55].

### 6.2. Efficacy and Functional Outcomes

In the 2025 network meta-analysis, single-portal ECTR produced the largest early functional gains and reduced symptom severity at one, three, and six months versus open CTR, and dual-portal ECTR demonstrated reduced early pain [25]. UGCTR did not significantly outperform open CTR on symptom severity or function, though these comparisons were rated as very low confidence [25].

### 6.3. Return to Work and Patient Satisfaction

Earlier return to work is the most reproducible benefit of minimally invasive CTR. The 2014 Cochrane review estimated return to work approximately 8 days earlier after endoscopic CTR, with greater early grip strength, on low- to very-low-certainty evidence [29]. For UGCTR, a 2026 meta-analysis reported return to work approximately 10 days earlier and resumption of normal activities approximately 21 days earlier than open techniques [56]. In the network meta-analysis, UGCTR showed the earliest return to work of any approach except mini-open CTR (SMD −3.99 versus open) [25]. UGCTR also yielded the highest patient satisfaction of any approach in the network meta-analysis (OR 6.89 versus open) [25].

### 6.4. Complications and Revision Rates

ECTR may carry a higher rate of transient neurapraxia, attributed to passing instruments through the carpal canal. However, rates of major complications do not differ significantly from open CTR, and permanent nerve injury is rare [29,57,58]. Wound- and scar-related complications are lower after ECTR than open CTR [29,57]. Single- and dual-portal ECTR perform comparably on safety and efficacy in indirect comparisons [29,57,58]. Pooled complication and reoperation rates for UGCTR were comparable to open CTR [25,56].

A propensity-matched database analysis (*n* = 43,865) found a higher 2-year reoperation rate after ECTR (RR 1.15) that converged to no significant difference beyond five years (number needed to treat to prevent one reoperation, 67 at two years vs. 473 beyond five), suggesting the early revision excess may not persist [59]. Longer-term data suggest a modest increase in revision after ECTR. A retrospective comparison (*n* = 4338) demonstrated a 2.96 times higher revision rate within 1 year following ECTR compared to open CTR (2.3% and 0.71% respectively) [60]. A Veterans Administration cohort (*n* = 103,455) confirmed a persistent increase in revision hazard after ECTR over up to 15 years of follow-up; however, the absolute difference at ten years was small (0.72%) [61]. At revision, reconstituted TCL and incomplete release were more common after ECTR, while nerve and overlying soft-tissue scarring were more common after open CTR [61]. It remains unclear whether this reflects technical limitations potentially mitigated by surgeon experience or an inherent constraint of endoscopic visualization.

### 6.5. Guideline Recommendations

Major guidelines differ on their recommended surgical technique. The 2014 European HANDGUIDE favors open CTR through a longitudinal, non-extended incision [62]. The panel did not endorse ECTR, citing the absence of unequivocal evidence for the superiority of any technique and prioritizing direct visualization of the surgical field [2].

The 2016 AAOS guideline stated that there was limited evidence supporting ECTR based on short-term benefits, including faster motor recovery, earlier return to work, and lower rates of wound and scar complications. The 2024 update, based on multiple high- and moderate-quality studies, upgraded this to a strong recommendation that patient-reported outcomes do not differ between mini-open and ECTR in the long term. However, the 2024 guideline also noted that ECTR may be associated with greater costs and that complication rates “may be higher than previously described,” citing emerging large-database studies on revision rates [60,63].

These differences reflect both the timing of each guideline’s evidence review and differing methodological approaches to grading, rather than genuine disagreement about the underlying data. Neither guideline had sufficient data on UGCTR to issue technique-specific recommendations, a notable gap given the growing clinical adoption of these approaches. The absence of long-term comparative data and the heterogeneity of UGCTR mechanisms further limit the ability of current guidelines to inform technique selection within this category.

## 7. Practical Considerations

### 7.1. Learning Curve and Safety

Published data on single-portal ECTR demonstrate decreasing rates of conversion to open from approximately 6% in the first post-fellowship year to ~1% thereafter [26,27]. By contrast, operator ultrasound proficiency is commonly the rate-limiting skill for UGCTR. Published case-number thresholds (e.g., 30 procedures for operators with prior musculoskeletal ultrasound experience, 50 for those without) should be interpreted in context. Prior experience with ultrasound-guided interventions, familiarity with carpal tunnel sonoanatomy, and structured cadaveric training are likely to shorten the learning curve substantially, whereas case volume alone is an incomplete proxy for competence [64]. For thread-loop CTR specifically, the learning curve has been estimated at 25 supervised cases for operators with prior ultrasound experience and approximately 50 for those without [65].

Retrograde cutting directs force away from at-risk structures, and built-in safety elements (balloon, hydrodissection, bladeless thread) may widen the margin of safety during the learning curve, whereas techniques relying solely on operator visualization demand a higher baseline ultrasound proficiency. Cadaveric training before independent practice is recommended for all UGCTR techniques.

### 7.2. Patient Selection and Contraindications

Beyond intraoperative guidance, preprocedural high-frequency ultrasound serves as a critical patient-selection tool. Systematic sonographic evaluation can identify bifid median nerves, persistent median arteries, transligamentous motor branch variants, and intratunnel space-occupying lesions that may contraindicate a percutaneous approach or prompt selection of a technique permitting direct visualization of the motor branch.

Common contraindications across minimally invasive CTR include intratunnel space-occupying lesions, revision CTR, acute carpal tunnel syndrome from median artery thrombosis or trauma, isolated motor-form carpal tunnel syndrome, and severe anatomic distortion from distal radius malunion or inflammatory arthritis [8,66]. Open or hybrid ultrasound-assisted CTR are often preferred for variants of the recurrent motor branch or Berrettini communicating branch [8,66]. For UGCTR, insufficient visualization, whether driven by body habitus, operator-equipment limits, or anatomic variation, may preclude percutaneous CTR [46,50,64].

Both retractable-blade and hook-knife classes have published data in patients with diabetes, anticoagulant use, severe electrophysiologic findings, and bifid median nerve anatomy, supporting feasibility across these higher-risk populations [40,67]. Certain neurological comorbidities also warrant consideration; carpal tunnel syndrome is more prevalent in populations such as patients with Parkinson’s disease, which may influence patient selection and counseling [68].

### 7.3. Office Feasibility and Anesthesia

The 2024 AAOS guideline endorses local anesthesia alone on the basis of high-quality evidence and office-based CTR on limited evidence [63]. Patient satisfaction studies show a clear preference for wide-awake local anesthesia no tourniquet (WALANT) over tourniquet-based intravenous regional anesthesia, with tourniquet pain the main source of dissatisfaction [9,69]. Proximal regional blocks (e.g., axillary or supraclavicular) can cover tourniquet pain but require additional resources and monitoring that may offset the office-based advantages of minimally invasive CTR. Distal nerve blocks at the wrist do not reliably cover a proximal arm tourniquet; combining them with WALANT eliminates tourniquet pain and reduces perioperative discomfort [70].

Importantly, ECTR relies on endoscopic visualization within the canal, making a tourniquet functionally important for a clear field, whereas UGCTR visualization is unaffected by intratunnel blood. This distinction has direct implications for office feasibility; UGCTR can be performed entirely under WALANT without compromising visualization, whereas ECTR typically requires tourniquet hemostasis to maintain an adequate endoscopic field.

### 7.4. Cost and Sustainability

From a payer or health-system standpoint, office-based open CTR is the most cost-effective option [71,72]. Facility costs were lower with WALANT than with monitored anesthesia care, by about 35% (roughly $170 per case) in a time-driven activity-based costing analysis [71]. ECTR is associated with higher costs than open CTR within any setting, including procedure rooms and operating rooms [72,73]. From a societal standpoint, cost-utility analyses demonstrated that UGCTR was the dominant strategy over both open and endoscopic CTR, primarily driven by earlier return-to-work [73,74]. Cost-effectiveness estimates are sensitive to local reimbursement structures, device pricing, and facility overhead, and findings from U.S.-based analyses may not generalize to other healthcare systems.

Office-based CTR has been demonstrated to reduce greenhouse gas emissions by approximately 60% compared to CTR in an operating room [75]. A separate analysis reported a larger carbon footprint for ECTR than open CTR, though this was driven partly by institution-specific sterilization processes and may not generalize [76]. The compatibility of UGCTR with office-based WALANT settings may confer additional cost and sustainability advantages, though this has not been formally modeled. Whether single-use devices (which add manufacturing and disposal waste) or reusable ones (which shift the burden to sterilization and reprocessing) are more sustainable remains unknown.

## 8. Limitations and Future Directions

Most comparative trials use open or mini-open CTR as the control; head-to-head trials between ECTR and UGCTR are limited to a single prospective propensity-matched cohort, with no randomized head-to-head data and none comparing the different UGCTR mechanisms. Many UGCTR series are single-operator or single-center, and industry sponsorship or inventor involvement is common in the device literature. Long-term comparative follow-up beyond two years remains available only for ECTR. Outcome measures are reported inconsistently, and a standardized definition of complete release has not been adopted, limiting cross-mechanism comparison. The most frequently reported outcome measures across included studies were the Boston Carpal Tunnel Questionnaire (symptom severity and functional status scales), QuickDASH, visual analog scale for pain, grip and pinch strength, nerve conduction parameters, and return-to-work interval, though definitions and reporting timepoints varied considerably. Device-specific registries with standardized outcome reporting and long-term follow-up will be essential to distinguish safety and efficacy profiles within UGCTR.

## 9. Conclusions

This review classified minimally invasive CTR into eight mechanistically distinct techniques, two endoscopic and six ultrasound-guided, each differing in transection mechanism, safety features, and evidence maturity. Office-based minimally invasive CTR offers demonstrated advantages in earlier return to work and reduced scar-related morbidity. However, balanced against the higher revision rate and higher costs associated with ECTR, the tradeoff remains unclear, as reflected in divergent guideline recommendations. UGCTR may offer similar advantages at lower cost and potentially lower revision rates; however, long-term data are lacking, and the core outcomes may differ between mechanisms given differences in safety features, learning curves, and evidence maturity. As these techniques evolve and migrate across specialties, this mechanism-based classification can facilitate direct comparisons and inform evidence-based adoption.

## Figures and Tables

**Table 1:** Summary of minimally invasive carpal tunnel release techniques classified by transection mechanism.

Class	Mechanism	Representative Devices	Incision	Cutting Direction	Safety Mechanism	Evidence Base ^1^
**Endoscopic**						
Single-portal	Integrated endoscope and retractable blade within a slotted cannula; TCL transected on device withdrawal	SmartRelease (MicroAire Surgical Instruments) and Agee-derived systems	1–2 cm transverse, wrist crease	Retrograde	Direct endoscopic visualization; slotted cannula constrains blade trajectory	NMA; multiple RCTs; pooled >40,000 cases; multicenter
Dual-portal	Endoscope and hook knife introduced through opposing proximal and distal portals; TCL transected under direct visualization	Chow technique (standard endoscopic instrumentation)	1 cm proximal (radial to pisiform) and distal mid-palmar	Retrograde	Bidirectional endoscopic visualization of the TCL from both portals	Multiple RCTs; NMA
**Ultrasound-guided**						
Retractable retrograde blade	Retractable blade within cannula advanced beneath TCL; balloon or hydrodissection displaces the median nerve before transection	SX-One MicroKnife/UltraGuideCTR (Sonex Health, Eagan, MN, USA); MICROi-Blade (Summit Medical Products, Inc, Sandy, UT, USA); MANOS CTR (Thayer Intellectual Property, Inc., San Francisco, CA, USA)	Sub-centimeter wrist crease or distal forearm; MANOS adds a distal palmar puncture	Retrograde	Saline-inflated balloon (UltraGuideCTR)	Multicenter RCT (*n* = 149); multicenter registry
Hook-knife	Curved hook blade advanced beneath TCL and rotated to engage ligament; transected by retrograde pull-through	3.0 mm Acufex hook knife (Smith & Nephew PLC, London, UK)	1–3 mm distal antebrachial	Retrograde	Hydrodissection; optional guiding cannula	Single-center RCT (*n* = 92); single-operator series
Thread-loop	Cutting thread looped around TCL via two needle passes; reciprocal sawing motion transects TCL	Loop&Shear (Ridge & Crest, Monterey Park, CA, USA); Smartwire (Ilsan, South Korea)	Incisionless (two needle punctures)	Bidirectional	Hydrodissection; bladeless thread	2 small RCTs (*n* = 11, *n* = 20); retrospective cohort (*n* = 82)
Antegrade blade	Curved blade applied to the proximal TCL edge and advanced distally to transect the TCL	KeriKnife (KeriMedical, Plan-les-Ouates, Switzerland); Kemis H3 (Newclip Technics, Haute-Goulaine, France)	Typically, a 7–15 mm wrist-crease incision	Antegrade	Hydrodissection; integrated cutting guide and distal stop element (KeriKnife); protective end tip (Kemis H3)	Cadaveric study; 1 prospective cohort (*n* = 50); no RCT
Pinhole scalpel	Small blunt-edged cutter introduced through catheter puncture; progressive antegrade cuts under mechanical tension	Sono-Instruments (Spirecut, AG, Muttenz, Switzerland)	Incisionless (14-gauge puncture)	Antegrade	Tension-selective mechanism	Single-surgeon prospective series (*n* = 30); no RCT
Needle-knife/acupotomy	Flat-bladed miniscalpel-needle advanced through TCL; multiple forward-and-backward passes transect the TCL	Miniscalpel-needle	Incisionless (needle puncture)	Antegrade	Operator ultrasound visualization	Multiple small single-center RCTs; meta-analyses

Abbreviations: CTR, carpal tunnel release; CTS, carpal tunnel syndrome; NMA, network meta-analysis; RCT, randomized controlled trial; TCL, transverse carpal ligament. ^1^ Evidence base reflects a narrative summary of the available literature for each technique and does not represent a formal systematic review or quality-of-evidence grading.

## Data Availability

No new data were created or analyzed in this study. Data sharing is not applicable to this article.

## Abbreviations

The following abbreviations are used in this manuscript:

AAOS	American Academy of Orthopaedic Surgeons
ASSH	American Society for Surgery of the Hand
CTR	Carpal tunnel release
ECTR	Endoscopic carpal tunnel release
MRI	Magnetic resonance imaging
NMA	Network meta-analysis
OR	Odds ratio
QuickDASH	Quick Disabilities of the Arm, Shoulder, and Hand
RCT	Randomized controlled trial
RR	Relative risk
SMD	Standardized mean difference
TCL	Transverse carpal ligament
UGCTR	Ultrasound-guided carpal tunnel release
